# Sunlight Effects on the Osmotrophic Uptake of DMSP-Sulfur and Leucine by Polar Phytoplankton

**DOI:** 10.1371/journal.pone.0045545

**Published:** 2012-09-19

**Authors:** Clara Ruiz-González, Martí Galí, Eva Sintes, Gerhard J. Herndl, Josep M. Gasol, Rafel Simó

**Affiliations:** 1 Department of Marine Biology, Institut de Ciències del Mar-CSIC, Barcelona, Spain; 2 Department of Marine Biology, Faculty Center of Ecology, University of Vienna, Vienna, Austria; Université Paris Sud, France

## Abstract

Even though the uptake and assimilation of organic compounds by phytoplankton has been long recognized, very little is still known about its potential ecological role in natural marine communities and whether it varies depending on the light regimes the algae experience. We combined measurements of size-fractionated assimilation of trace additions of ^3^H-leucine and ^35^S-dimethylsulfoniopropionate (DMSP) with microautoradiography to assess the extent and relevance of osmoheterotrophy in summer phytoplankton assemblages from Arctic and Antarctic waters, and the role of solar radiation on it was further investigated by exposing samples to different radiation spectra. Significant assimilation of both substrates occurred in the size fraction containing most phytoplankton (>5 µm), sunlight exposure generally increasing ^35^S-DMSP-sulfur assimilation and decreasing ^3^H-leucine assimilation. Microautoradiography revealed that the capacity to take up both organic substrates seemed widespread among different polar algal phyla, particularly in pennate and centric diatoms, and photosynthetic dinoflagellates. Image analysis of the microautoradiograms showed for the first time interspecific variability in the uptakes of ^35^S-DMSP and ^3^H-leucine by phytoplankton depending on the solar spectrum. Overall, these results suggest that the role of polar phytoplankton in the utilization of labile dissolved organic matter may be significant under certain conditions and further confirm the relevance of solar radiation in regulating heterotrophy in the pelagic ocean.

## Introduction

The ability to take up and utilize dissolved organic matter (DOM) as a source of carbon and energy (hereafter ‘osmoheterotrophy’) was demonstrated for a wide variety of algal cultures more than three decades ago (see refs. in [1], [2], [3]), yet it was initially thought to be ecologically irrelevant due to the inability of the algae to compete with bacteria at the low substrate concentrations found in natural environments [4], [5], [6]. Owing to their high surface to volume ratio and their efficient uptake systems, heterotrophic bacteria are regarded as the most efficient consumers of DOM [5], and consequently, phytoplankton osmoheterotrophy is neglected in most geochemical models of carbon flow [7], [8].

However, some studies have shown that several phytoplankton species do actively take up substrates at low concentrations so that they may, in fact, be competitive with bacteria [9], [10]. Among the organic substrates algae are able to use are pyruvate, acetate, lactate, ethanol, saturated fatty acids, glycolate, glycerol, hexoses, urea, and amino acids (e.g. [11], [12], [13], [14]). More recently, it was discovered that a variety of marine phytoplankton taxa can also take up the ubiquitous algal synthate dimethylsulfoniopropionate (DMSP) [15], [16] and assimilate its sulfur [15], thus influencing the cycling of organic sulfur in the surface ocean. These evidences, together with the phagotrophy described for many algal groups [17], [18], suggest that algae may play a more diverse role in aquatic biogeochemical cycles than just supplying heterotrophs with autotrophically synthesized organic matter. Moreover, while most studies on phytoplankton osmoheterotrophy have focused on algal cultures (which may not be representative of ecologically relevant organisms) and on freshwater or benthic systems, pelagic marine environments have received less attention and very little is known about the role of algal osmoheterotrophy in natural marine communities.

The uptake and assimilation of organic substrates by algae often increase with decreasing light availability [19], [20], although either enhanced uptake under light exposure [21], [22], [23] or no effect of irradiance on uptake rates [24], [25] have also been reported. Studies of algal osmoheterotrophy have commonly exposed cells to artificial light, and although some have considered in situ light conditions, to our knowledge none has specifically assessed the effect of natural solar ultraviolet radiation (UVR, 280–400 nm).

Research on the effects of UVR (and mainly UVB, 280–320 nm) on aquatic food webs has gained increasing attention, and this has been particularly so in the polar regions, where there is evidence that ozone depletion [26], [27] and the ongoing loss of sea-ice [28], [29] are leading to enhanced underwater levels of UVR. In these regions, the continuous darkness during the polar winter, the low irradiance under the sea ice layer and the relatively high concentrations of labile organic nutrients [30], [31] may select for algae with heterotrophic or photoheterotrophic capabilities. As an example, Rivkin and Putt [21] found that Antarctic algae incorporated amino acids and glucose at ambient concentrations and proposed that this ecological trait might supplement light-limited growth during the polar spring and summer as well as support heterotrophic growth throughout the polar winter.

The first aim of this work was to assess the occurrence and relevance of the utilization of dissolved organic compounds in natural marine phytoplankton assemblages during the Arctic and Antarctic summers by tracking the fate of two ubiquitous low-molecular-weight (LMW) dissolved organic compounds: leucine and DMSP. Our second aim was to address the effect of natural solar radiation on the uptake of these compounds by different phytoplankton groups. We combined measurements of size-fractionated radioisotope uptake and assimilation with a microautoradiographic approach to identify the organisms taking up the respective radiolabeled substrate. Image analysis of microautoradiograms allowed to determine group-specific substrate affinities and sensitivities to UVR. This study relates to our previous work on sunlight effects on the assimilation of these substrates by major bacterial groups in the same communities [32].

## Methods

### Study Area and Sample Collection

**Figure 1:**
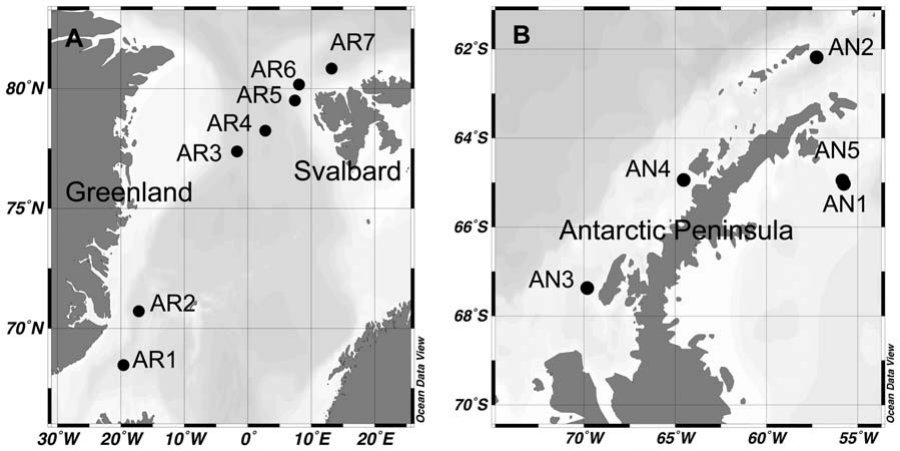
Map of the stations where different experiments were performed. (A) Arctic stations, July 2007; (B) Antarctic stations, February 2009. Maps were generated with the Ocean Data View software (http://odv.awi.de).

The study was carried out on board RV Hespérides during the ATOS I and II cruises to the Arctic and Antarctica (Fig. 1). In July 2007, ATOS I visited the Atlantic sector of the Arctic with a transect from Iceland, parallel to the eastern Greenland current, up to the ice cap edge (*ca.* 81°N) located north/northwest of Svalbard. In February 2009, ATOS II cruised around the Antarctic Peninsula, from the Weddell Sea (65°S) through the Bransfield Strait and into the Bellingshausen Sea (*ca.* 69°S). No specific permits were required for the described field studies. In Antarctic waters, the activities conducted met the requirements and protocols of the Antarctic Treaty as reviewed and approved by the Spanish Polar Committee. The locations of study were not privately-owned nor protected beyond the Antarctic Treaty, and the field studies did not involve endangered or protected species.

Samples for size-fractionated uptake and assimilation measurements and for microautoradiography were collected at 5 m depth (except for station AN2, sampled at 20 m depth, where high abundances of the diatom *Pseudonitzschia* were found) with a rosette of Niskin bottles mounted on a CTD profiler. Water characteristics of the sampled stations together with the irradiance measurements and time of incubation during experiments are compiled in Table 1.

**Table 1:** Sampling stations and experimental conditions.

Arctic	Stn	Date (day/mo/yr)	Longitude	Latitude	SW Temp (°C)	Salinity (PSU)	Sampling depth (m)	Incub. time(h)	PAR(E m^−2^)	UVA(kJ m^−2^)	UVB (kJ m^−2^)
	AR1	01/07/07	68° 28.8′W	19° 30.3′N	2.41	32.5	5	8.8	–	–	–
	AR2	02/07/07	17° 08.2′W	70° 43.3′N	−0.08	33.4	5	10	–	–	–
**	AR3	05/07/07	1° 39.8′W	77° 23.2′N	3.22	34.3	5	12	11.1	272.8	9.2
**	AR4	07/07/07	2°51.5′E	78°13.8′N	2.15	33.6	5	11.5	5.4	140.8	4.6
*	AR5	12/07/07	7° 29.6′E	79° 30.1′N	0.15	32.4	5	11.8	11.4	244.5	7.4
*	AR6	14/07/07	8° 05.2′E	80° 09.9′N	1.36	32.8	5	10	10.0	221.1	6.7
*	AR7	19/07/07	13° 14.2′ E	80° 49.6′N	0.21	31.9	5	9.5	5.3	134.7	4.5
Antarctica											
**	AN1	03/02/09	55° 45.4′W	65° 01.2′S	−0.17	27.9	5	7.6	8.4	215.9	9.1
**	AN2	06/02/09	57° 14.4′W	62° 10.6′S	1.67	29.9	20	8	1.3	34.8	1.2
	AN3	19/02/09	69° 48.4′W	67° 22.3′S	1.44	26.9	5	7.5	–	–	–
	AN4	21/02/09	64° 32.2′W	64° 56.5′S	2.46	30.3	5	8.1	–	–	–
	AN5	25/02/09	55° 50.0′W	64° 57.0′S	−0.73	27.6	5	7.2	–	–	–

Characteristics of the different stations sampled for dark size-fractionated assimilation measurements, time of incubation during experiments and radiation doses received by samples in which light experiments were performed.

(*) Stations where samples for size-fractionated assimilation were also incubated under different light conditions.

(**) Stations where, besides size-fractionated assimilation measurements, incubations for microautoradiographic analysis were carried out.

### Experimental Design

We performed a number of experiments (seven for size fractionated assimilation and four for microautoradiography, see below) to assess the impact of natural solar radiation on the heterotrophic activity of polar microalgae. Briefly, water samples were incubated in UVR-transparent quartz bottles amended with trace concentrations of ^35^S-DMSP (donated by R. P. Kiene, University of South Alabama, Dauphin Island Sea Lab, USA) or ^3^H-leucine (Amersham) under different light conditions. Bottles were either exposed to the full solar radiation spectrum (PAR+UVR), the full spectrum without UVB (i.e., PAR+UVA, covered with Mylar-D foil) or kept in the dark. Samples were incubated inside a black tank with running seawater to maintain *in situ* temperature. To simulate the irradiance level of 5 m depth, samples were placed 5 cm under the surface below an optically neutral mesh that reduced surface irradiances by 40%. Samples from station AN2 (20 m depth) were covered with a double neutral mesh that reduced surface irradiances by 60%.

### Radiation Measurements

UVR and PAR radiation inside the incubation tank were continuously monitored throughout the incubations with a Biospherical PUV-radiometer 2500. The downwelling cosine irradiance reaching the samples was recorded at a frequency of 5 s^−1^. The wavelengths measured included one integrated band in the visible (PAR, 400–700 nm, *µ*mol photons cm^−2^ s^−1^) and 6 channels within the UVR range (305, 315, 320, 340, 380, 395 nm, in mW cm^−2^ nm^−1^). The mean spectral irradiance in the 6 UVR bands was converted to mean UVB and UVA irradiance (mW cm^−2^) by integrating over the spectrum (sum of trapezoids), between 305–320 nm and 320–395 nm, respectively. Finally, the mean UVB, UVA and PAR irradiance was multiplied by the duration of each experiment in order to obtain the radiation dose (in kJ m^−2^ for UVB and UVA, and mol photons m^−2^ for PAR), shown for each experiment in Table 1.

### Size Fractionated Assimilation

Samples of 50 ml were incubated for 7 to 12 h in quartz bottles with added trace concentrations of ^35^S-DMSP (845 Ci mmol^−1^, 0.8 pM final conc. for Arctic samples and 120–145 Ci mmol^−1^, 2.5–3 pM final conc. for Antarctic samples) or ^3^H-leucine (161 Ci mmol^−1^, 0.5 nM final conc.). Controls were killed with paraformaldehyde (PFA, 1% final conc.) before the addition of the radioactive compound and were exposed to the same conditions as live samples. After exposure, the incorporation of substrate was stopped by overnight PFA-fixation (1% final conc.) at 4°C in the dark, and duplicate or triplicate subsamples of 15–25 ml were filtered through 5 µm pore-sized filters (SMWP, Millipore); the filtrate was subsequently filtered through 0.2 µm pore-sized filters (GNWP, Millipore) and rinsed with 0.2 µm filtered seawater. The fraction collected on 0.2 µm filters was mainly comprised by prokaryotic cells, as revealed by microscopy. With the filtration system off, macromolecules were precipitated by pouring 5 ml of cold 5% trichloroacetic acid (TCA) onto the filters for 5 min. Then, the TCA was removed and the filters were rinsed with Milli-Q water. Radioactivity was determined by placing them into 5 ml of scintillation cocktail (Optimal HiSafe) and counting with a Beckman scintillation counter.

### Microautoradiography of Algae

Samples of 50 ml were incubated under the different light treatments with added ^35^S-DMSP (845 Ci mmol^−1^, 0.04 nM final conc. for Arctic samples and 145 Ci mmol^−1^, 0.03 nM final conc. for Antarctic samples) or ^3^H-leucine (161 Ci mmol^−1^, 0.5 nM final conc.) for 7 to 12 h. Controls killed with PFA were also run simultaneously with all live incubations. After sunlight exposure, live samples were fixed overnight with PFA (1% final conc.) at 4°C in the dark. Aliquots of 15–20 ml were gently filtered through 5 µm polycarbonate filters (Osmonics, inc.), rinsed with Milli-Q water, air dried and stored at −20°C until processing. Microautoradiography of ^35^S-DMSP samples from station AN2 could not be performed due to an insufficient amount of the radiolabeled substrate used.

Microautoradiography was carried out as described by Vila-Costa et al. [15]. Filters were developed after 6 d for ^3^H-leucine and 18 d for ^35^S-DMSP in Arctic samples, and 20 d for ^3^H-leucine and 2 mo for ^35^S-DMSP in Antarctic samples, and stained with 4,6-diamidino-2-phenylindole (DAPI, 1 µg ml^−1^). Labeled cells were counted under an Olympus BX61 epifluorescence microscope within the major groups showing consistent uptake of any of the substrates. Active and inactive cells were clearly distinguished by the presence or absence of silver grain accumulations denser than the background. Between 30 and 700 cells were considered for obtaining the percentages of active cells. Epifluorescence microscopy combined with scanning electronic microscopy was used to identify the eukaryotic microorganisms present in our samples.

### Image Analysis of the Silver Grain Area Surrounding Active Algal Cells

We followed the protocol described by Sintes and Herndl [33] with some modifications for algal images. For each individual cell, three images were acquired: one of the alga stained with DAPI, one of the fluorescence of chlorophyll (both in epifluorescence mode of a Zeiss Axioplan 2 microscope) and a third image of the silver grains by switching to the transmission mode of the microscope. The images were acquired with a digital camera (AxioCam MRc5) mounted on the microscope. Pictures were taken of 20 to 60 cells per phytoplankton group and treatment. Overlapping signals in the DAPI + chlorophyll images and the transmitted light images (silver grains) indicated cells that had assimilated ^35^S from DMSP or ^3^H from leucine. Image analyses were conducted with the KS300 3.0 software (Carl Zeiss), which allowed us to record the area of each cell, as well as the silver grain area around it. Several, but not all the algal groups were considered for this analysis. In the Arctic experiments, the five groups analyzed were two pennate diatoms (*Pseudonitzschia* spp., *Navicula* spp.), a group of centric diatoms (*Thalassiosira* spp.), photosynthetic dinoflagellates (mainly *Prorocentrum* spp., although other species were also included), and the dominant flagellate *Phaeocystis* sp. From Antarctic waters (Station AN1), *Pseudonitzschia* spp., three different species of *Thalassiosira* (spp. A, B and C) with distinct size and chloroplast distribution, and a group of unidentified heterotrophic nanoflagellates were considered. The latter were the only heterotrophic organism analyzed, as they were the ones showing by far the largest ^35^S-silver grain areas at that station. For background correction, three pictures from each filter were taken of an area containing no cells, and the averaged silver grain area per background area was subtracted from the average silver grain area per cell area measured for each algal group. ^3^H-leucine samples from station AR3 and AN2 were not analyzed since too few cells were labeled.

Additionally, DAPI-stained bacteria retained onto these filters were counted in order to estimate their contribution to apparent algal substrate uptake. Since many occurred on aggregates, silver grains could not be attributed to individual bacteria, but an estimated mean silver grain area per bacterial cell was obtained by dividing the silver grain area by the number of bacterial cells in the aggregate.

### Statistical Analyses

Shapiro-Wilk’s *W*-test for normality of data and Levene’s test for homogeneity of variance were applied prior to analysis, and either ANOVA or the nonparametric Kruskal-Wallis test was used to address statistically significant differences (*p*<0.05) in the measured variables. Post hoc analyses (Tukey’s test) were applied for comparison among different light treatments. Correlations between variables were calculated using the Pearson’s correlation coefficient. These statistical analyses were performed using the JMP software (SAS Institute).

## Results

### Background Information

The waters sampled during the two cruises displayed varying temperature (range −0.73–3.22°C) and salinity values (range 26.9–34.3) depending on the influence of the ice-melting (Table 1). While in Arctic waters there was a prevalence of the flagellate *Phaeocystis* sp., diatoms were the dominant phytoplankton in Antarctic stations. The four stations selected for microautoradiographic analyses from both cruises displayed elevated phytoplankton abundances typical of summer blooms.

### Bulk Assimilation of ^3^H-leucine and ^35^S-DMSP by Size-fractionated Plankton

**Figure 2:**
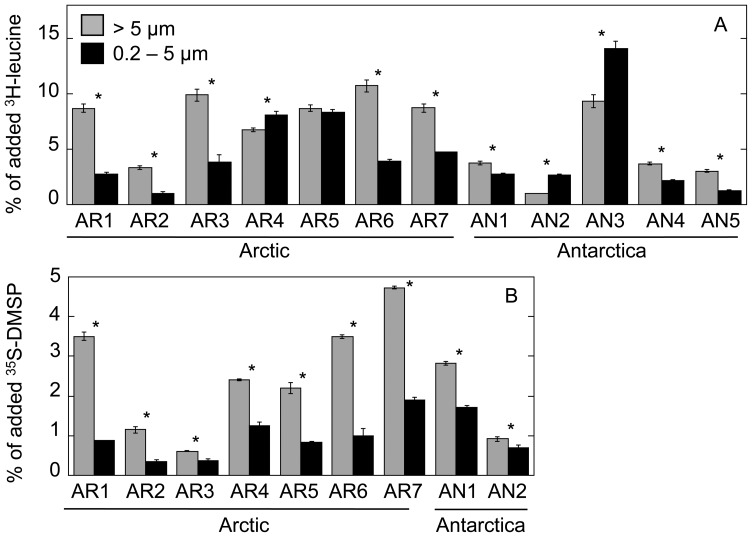
‘Algal’ vs. ‘bacterial’ assimilation of ^3^H-leucine and ^35^S-DMSP. Comparison between the percentages of assimilated ^3^H-leucine (A) and ^35^S-DMSP (B) of total added substrate by organisms >5 µm (grey bars) and 0.2–5 µm organisms (black bars) measured at different stations (average ± standard errors). All incubations were performed in the dark. Asterisks (*) indicate significant differences between both fractions (ANOVA, *p*<0.05).

The assimilation of both ^3^H-leucine and ^35^S-DMSP by differently sized microorganisms was measured by fractionating the samples through 5 µm and 0.2 µm filters after incubation with these radiotracers under different light spectra. For a general view, the results of the dark incubations are presented in Figure 2. While leucine assimilation by organisms >5 µm was significantly higher than in the size fraction 0.2–5 µm in 8 out of 12 stations (*p*<0.05, Fig. 2A), assimilation of ^35^S-DMSP was always significantly lower in the 0.2–5 µm fraction (Fig. 2B), which mainly comprised heterotrophic bacteria.

### Sunlight Effects on Substrate Assimilation by Organisms >5 µm

**Figure 3:**
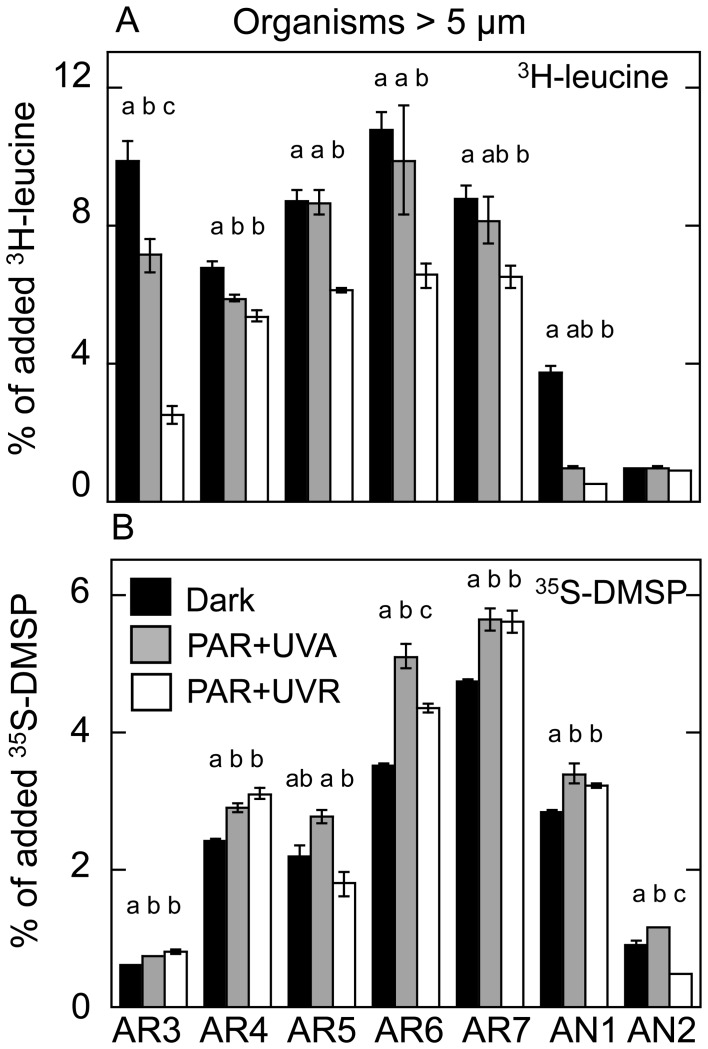
Effects of solar radiation on substrate assimilation. Percentages of assimilated ^3^H-leucine (A) and ^35^S-DMSP (B) of total added substrate by organisms >5 µm measured after exposure to the following radiation conditions: PAR+UVA (dashed bars), PAR+UVR (white bars) and darkness (black bars). Values are averages ± standard errors. Letters refer to results with a post hoc Tukey’s test. Different letters indicate significant differences (*p*<0.05) among treatments.

**Figure 4:**
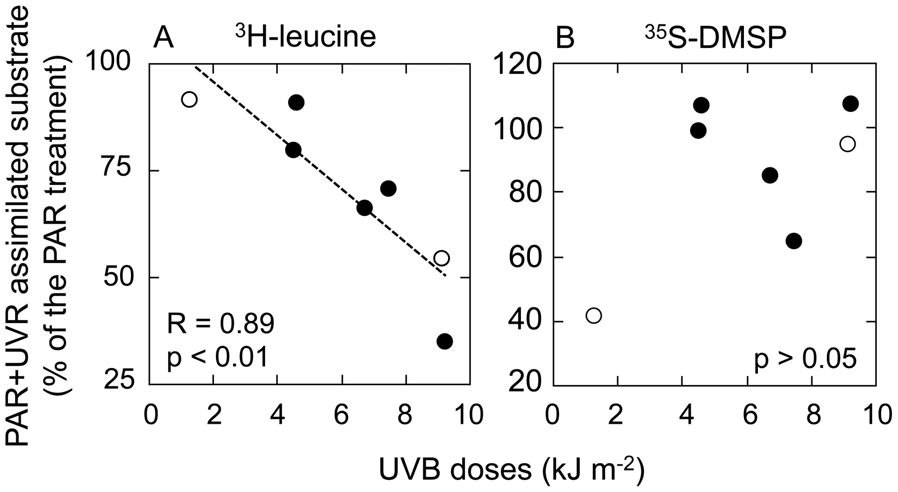
UVB doses versus inhibition of substrate incorporation. Relationships between the UVB-driven changes in the incorporation of (A) ^3^H-leucine or (B) ^35^S-DMSP and the UVB doses received by Arctic (black circles) and Antarctic (open circles) samples during each experiment.

The results of the subset of incubations performed also under PAR and UVR light are presented in Fig. 3. Exposure to the full spectrum of solar radiation (including UVB) caused a significant (Tukey’s test, *p*<0.05) reduction of ^3^H-leucine assimilation by organisms >5 µm compared to dark treatments at all stations except AN2 (range 20% to 75% decrease, Fig. 3A). Removal of UVB from the solar spectrum yielded higher assimilation percentages, yet never exceeding those of the dark controls. Interestingly, this variability in UVB-induced inhibition of ^3^H-leucine uptake was significantly correlated with the measured UVB doses during experiments (Pearson’s *r* = 0.88 and *r = *0.89, *p*<0.01, n = 7, compared to dark and PAR+UVA (Fig. 4A) treatments, respectively). Conversely, assimilation of ^35^S-DMSP by organisms >5 µm were consistently stimulated by light exposure (Fig. 3B), showing up to 33% and 45% increases due to PAR+UVA and full sunlight exposure, respectively, compared to the dark control (*p*<0.05). At three stations (AR5, AR6 and AN2), the full spectrum of solar radiation significantly inhibited the uptake of DMSP compared to PAR+UVA (Fig. 3B) but none of these responses appeared to be directly related to the light doses (Fig. 4B).

### Differential Assimilation of ^35^S-DMSP and ^3^H-leucine by Phytoplankton Taxa

Microautoradiography was applied to 5 µm filters from four selected stations (AR3, AR4, AN1 and AN2) to determine which phytoplankton phyla, if any, were responsible for the detected assimilation of leucine and DMSP. We observed that in most stations ^35^S-DMSP and ^3^H-leucine uptake was widespread among diverse algal groups (Table 2). Percentages of active cells were obtained combining the three light treatments, since generally it was not the number of active algae, but the size of the silver grain area, which differed significantly among the light treatments. Only some groups displayed significantly different numbers of active cells among treatments (*p*<0.05), written in bold in Table 2: while *Phaeocystis* spp. (AR3 and AR4) and photosynthetic dinoflagellates (AR3) exhibited higher numbers of active cells in ^35^S-DMSP uptake upon dark incubation compared to the light treatments, *Pseudonitzschia* spp. from stations AR3 and AN1 showed higher uptake after PAR+UVA or both light treatments, respectively (*p*<0.05). Instead, ^3^H-leucine uptake by *Pseudonitzschia* spp. from AN1 was inhibited by light incubation. Estimates of abundances and ranges of cell areas within each group are also indicated in Table 2.

**Table 2:** Most common microorganisms identified at the different stations.

	Percentage of cells active in substrate uptake														
	Arctic								Antarctica						
	Stn. AR3				Stn. AR4				Stn. AN1				Stn. AN2		
	Cells ml^−1^	Size (µm^2^)	DMSP (%)	Leu (%)	Cells ml^−1^	Size (µm^2^)	DMSP (%)	Leu (%)	Cells ml^−1^	Size (µm^2^)	DMSP (%)	Leu (%)	Cells ml^−1^	Size (µm^2^)	Leu(%)
*Pseudonitzschia* spp.	180	21–69	**87±7**	26±5	1420	28–100	91±3	53±7	270	36–357	**57±47**	**9±2**	2350	113–239	1±1
*Navicula* spp.	135	102–157	98±2	0	90	83–190	98±1	0	–	–	–	–	–	–	–
*Lennoxia* spp.	395	110–170	98±2	0	495	179–183	100	0	–	–	–	–	–	–	–
*Thalassiosira* sp. A	110	57–391	60±5	38±3	190	61–242	94±1	46±2	4360	80–248	59±3	32±2	120	70–254	32±4
*Thalassiosira* sp. B	–	–	–	–	–	–	–	–	840	198–472	95±2	40±2			
*Thalassiosira* sp. C	–	–	–	–	–	–	–	–	220	876–2473	70±8	100	–	–	–
*Thalassiosira* sp. D	–	–	–	–	–	–	–	–	15	2371–5711	74±7	100	–	–	–
*Chaetoceros* spp.	45	118–150	44±2	50±4	55	63–174	17±3	47±4	570	57–943	11±1	43±2	–	–	–
*Fragilariopsis* spp.	80	5–12	0	0	170	6–13	0	0	530	13–28	0	21±4	–	–	–
*Eucampia* spp.	25	143–595	100	0	160	277–347	100	0	20	2355–3005	0	100	–	–	–
*Lithodesmium* spp.	–	–	–	–	–	–	–	–	7	5521–6214	91±5	74±2	–	–	–
*Corethron* spp.	–	–	–	–	–	–	–	–	3	3200–6300	0	100	<1	5500–7500	100
Unid. photosynt.dinoflagellates	460	56–300	**47±20**	0	145	61–877	68±12	0	–	–	–	–	–	–	–
Unid. heterotrophicdinoflagellates	180	96–370	98±2	2±1	160	168–440	98±2	0	175	112–314	42±5	0	225	40–148	4±0
*Protoperidinium bipes*	5	203–380	100	0	20	111–597	100	0	–	–	–	–	–	–	–
*Leucocryptos marina*	–	–	–	–	90	67–141	100	0	–	–	–	–	–	–	–
*Phaeocystis* spp.	8320	11–50	**4±4**	0	1940	16–56	**11±9**	0	–	–	–	–	–	–	–
Photosynt. nanoflagellates<10 µm	2250	5–24	87±10	2±2	550	6–32	95±1	0	270	13–25	12±2	5±2	670	4–13	0
Heterotr. nanoflagellates<10 µm	nd	nd	nd	nd	nd	nd	nd	nd	600	13–54	100	98±2	nd	nd	nd

Microscopic estimates of cell abundances, mean phytoplankton cell sizes (expressed as cell area), and percentage of cells showing uptake of ^3^H-leucine (Leu) or ^35^S-DMSP (average ± standard error of the three treatments, ‘*nd’,* not determined). Data in **bold** (averages ± standard error of the three treatments) indicate that significant differences (Tukey’s test *p*<0.05) were detected among the three light treatments (see Results). *Thalassiosira* sp. A was so named to differentiate it from species B, C and D from station AN1, but sp. A from different stations was probably not the same species.

At the two Arctic stations (AR3 and AR4) the flagellate *Phaeocystis* sp. dominated the phytoplankton assemblage (Table 2, see also [34]), co-occurring with both photosynthetic (mainly *Prorocentrum* spp.) and heterotrophic dinoflagellates (AR3), or pennate and centric diatoms (AR4, where *Pseudonitzschia* spp. was nearly as abundant as *Phaeocystis* sp.). Except the dominant *Phaeocystis* sp., which hardly ever appeared labeled, most of the analyzed groups showed high uptake of either one or both substrates, although the majority exhibited more cells active in ^35^S-DMSP uptake (Table 2). Clearly defined silver grain areas around cells were found upon incubation with ^35^S-DMSP, whereas the label for ^3^H-leucine was generally restricted to fewer Arctic groups.

**Figure 5:**
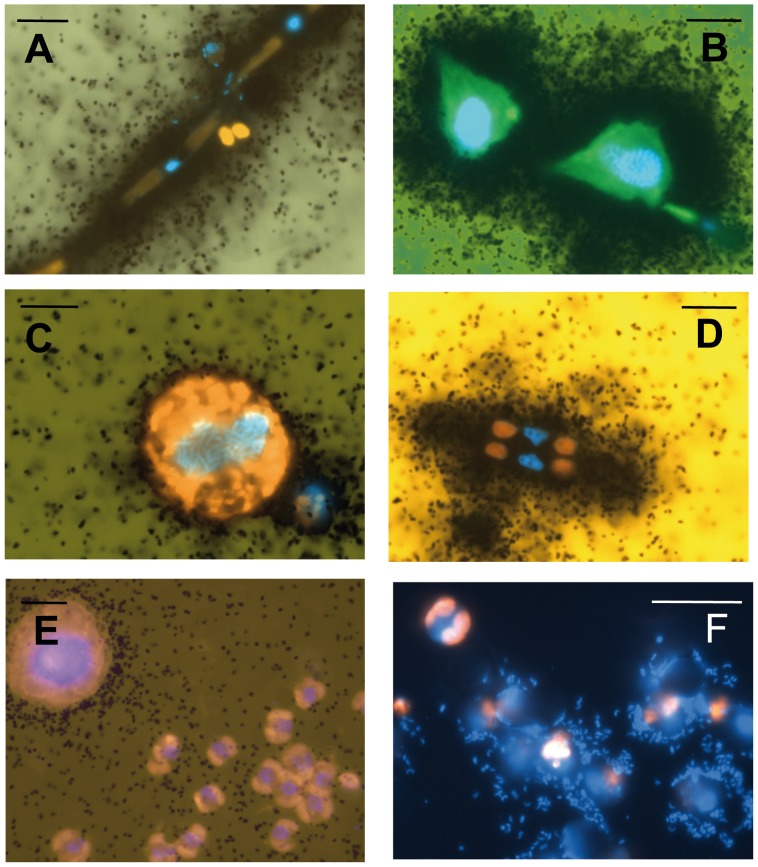
Microautoradiograms showing uptake of ^35^S-DMSP by different planktonic organisms in Arctic samples. (A) *Pseudonitzschia* sp.; (B) *Protoperidinium* sp.; (C) unidentified photosynthetic dinoflagellate; (D) *Navicula* sp. (E) unlabelled *Phaeocystis* sp. Black dots surrounding cells indicate uptake of the radioactive substrate by algae. (F) Clusters of well-localized silver grains occurred in association with single or clumped bacteria attached to *Phaeocystis* mucus and thus retained onto the 5 µm filters. Scale bar represents 10 µm.

Some typical ^35^S-DMSP autoradiograms from the Arctic samples are shown in Fig. 5. Diatoms such as *Pseudonitzschia* spp. (Fig. 5A) or *Navicula* (Fig. 5D) appeared intensely labeled. Clusters of well-localized silver grains also occurred in association with single or clumped bacteria that were attached to *Phaeocystis* mucus and thus were retained on the 5 µm filters (Fig. 5F). In all cases, prokaryotic cells were clearly visible due to DAPI staining and few bacteria adhering to live microalgae were sometimes present. For the enumeration of DMSP- or leucine-positive algae, only those devoid of attached bacteria were counted. Negligible numbers of labeled algal cells (<1%) were found in the killed controls.

Remarkably, the flagellate *Phaeocystis* sp., the dominant bloom former and DMSP producer in these waters, was only slightly labeled with ^35^S in the dark, but the signal was much weaker than that of the other algal groups (Fig. 5E).

**Figure 6:**
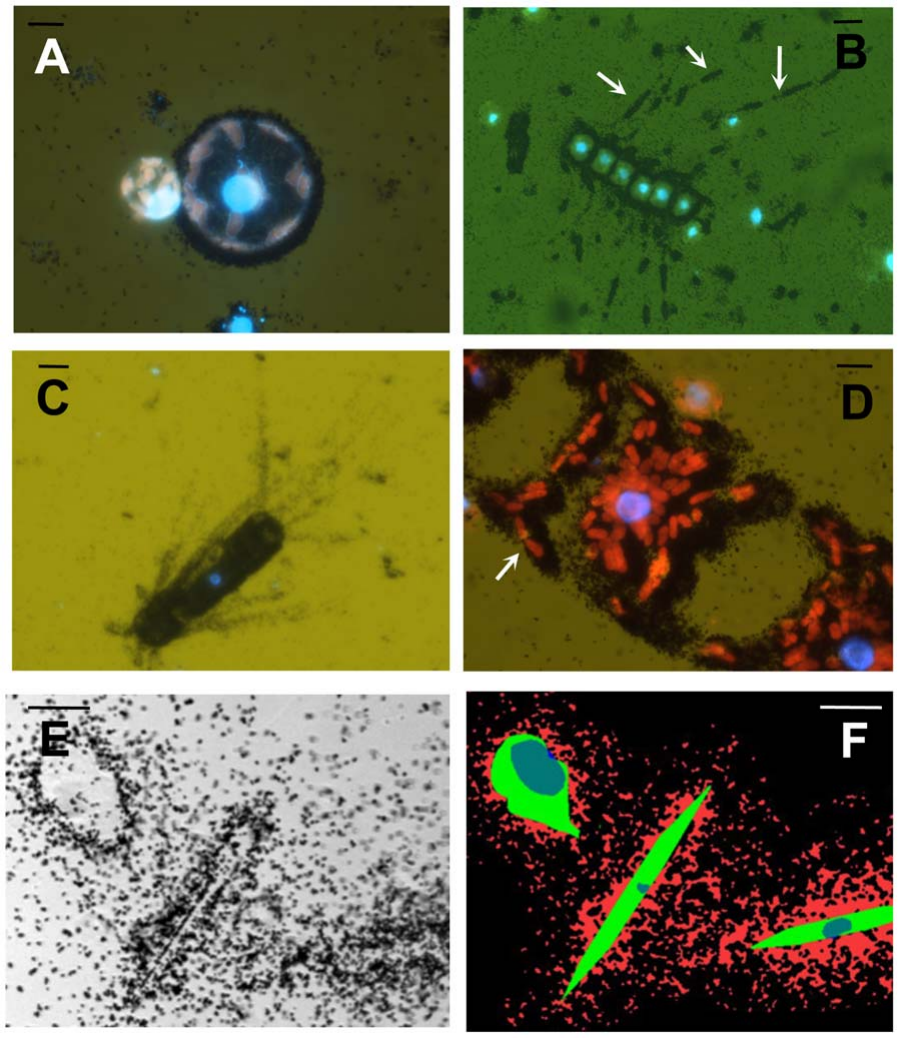
Microautoradiograms showing uptake of ^3^H-leucine by different planktonic algae in Antarctic samples. (A) Unlabelled *Thalassiosira* sp.B (left) and sp.C (right).; (B) *Chaetoceros* sp.; (C) *Corethron* sp.; (D) *Eucampia* sp. Note that the isotope seems to be specifically incorporated in structures such as chloroplasts (arrows in B and D). Scale bar represents 10 µm. (E and F) Examples of image analysis process. Three cells, shown under transmitted light in the left are digitized and nucleus, cell area and silver grain regions are identified, marked with different colours, and then sized (see Methods). Scale bar represents 10 µm.

The Antarctic stations AN1 and AN2 strongly differed in their phytoplankton composition. While a large number of *Thalassiosira-*like centric diatoms were found at station AN1, station AN2 was almost completely dominated by *Pseudonitzschia* spp. (Table 2). Microautoradiography was performed with both ^35^S-DMSP and ^3^H-leucine on samples of station AN1, while at station AN2, only ^3^H-leucine was used. In contrast to what was found for Arctic algae, ^35^S-DMSP silver grain areas were small for most groups at AN1 except for the heavily labeled small heterotrophic nanoflagellates. The large diatoms (*Chaetoceros* spp., *Eucampia* spp., *Lithodesmium* spp., *Corethron* spp. and some big *Thalassiosira* [spp. C and D]) showed clear preference for leucine over DMSP as illustrated by the high numbers of active cells (Table 2) and dense silver grain areas around them. Examples of leucine microautoradiograms of Antarctic algae are shown in Fig. 6. At station AN2, however, most of the radiolabel was associated with heterotrophic bacteria. Barely any of the dominant *Pseudonitzschia*, but just a few centric diatoms and heterotrophic dinoflagellates seemed to take up leucine.

### Differential Sensitivity to Solar Radiation among Phytoplankton Groups

**Figure 7:**
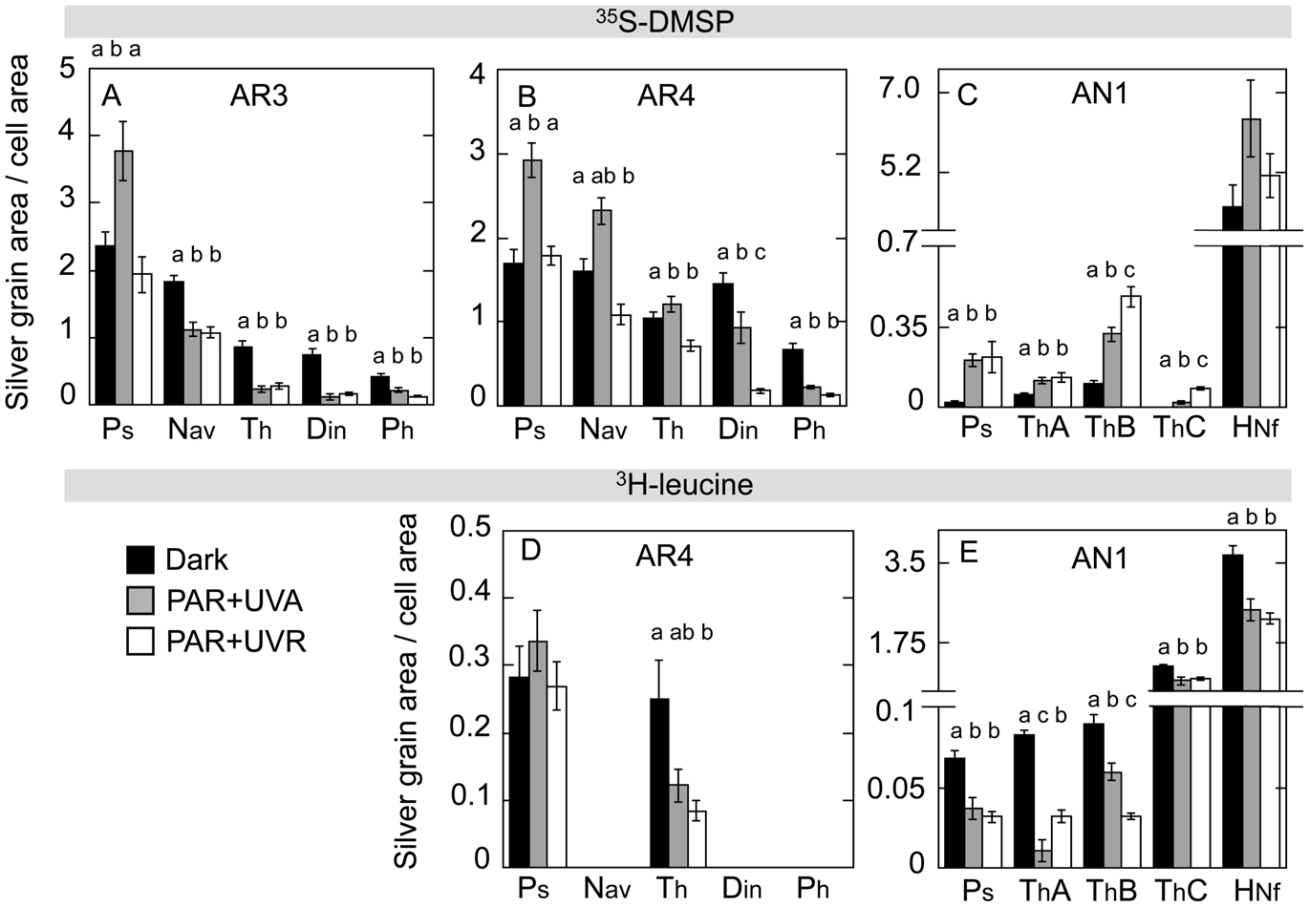
Sunlight effects on group-specific assimilation of ^3^H-leucine and ^35^S-DMSP. Average silver grain area per active algal cell in ^35^S-DMSP or ^3^H-leucine uptake as measured by image analysis of microautoradiograms (average ± standard error of 20 to 60 single cells) in 5 µm filters. Stn. AR3, AR4 and AN1, ^35^S-DMSP samples (A, B and C); Stn. AR4 and AN1, ^3^H-leucine samples (D and E). Samples were incubated under the following radiation conditions: PAR+UVA (dashed bars), PAR+UV (white bars) and darkness (black bars). Note the break in the Y axes in figures C and D. [Ps] *Pseudonitzschia* spp; [Nav] *Navicula* spp.; [ThA,B,C] *Thalassiosira* spp.A, B, C; [Din] Photosynthetic dinoflagellates; [Ph] *Phaeocystis* spp. [HNf] Heterotrophic nanoflagellates. Letters refer to results with a post hoc Tukey’s test. Different letters indicate significant differences (*p*<0.05) among treatments.

Microautoradiograms of 5 µm filters were subjected to image analysis to provide insight into the effects of sunlight on the uptake of these two organic compounds by some common groups of phytoplankton. Unfortunately, neither all groups nor all samples could be analyzed due to size or abundance limitations. In spite of the methodological uncertainties (see Discussion), we found group-specific responses to either substrate or light (Fig. 7). The pennate diatom *Pseudonitzschia* spp. from the Arctic, the one with the largest silver grain area per cell for ^35^S-DMSP, showed a significant increase (60% and 72% at Stn. AR3 and AR4, respectively, *p*<0.05) in the uptake of DMSP when exposed to PAR+UVA as compared to dark and full solar radiation conditions. Interestingly, although microautoradiograms from station AN2 are not available, the ^35^S-DMSP assimilated by the largest fraction, almost entirely comprised by *Pseudonitzschia* spp., also exhibited highest DMSP uptake in the PAR+UVA treatment (Fig. 3B). *Navicula* spp. at station AR4 also showed this pattern. In contrast, both light treatments significantly inhibited their ^35^S-DMSP uptake at station AR3. Generally, dark conditions seemed to stimulate ^35^S incorporation in the rest of the algal groups from station AR3 and AR4 (Figs. 7A and 7B), while most organisms from AN1 showed a significant photostimulation of ^35^S-DMSP uptake caused by both light treatments (Fig. 7C, *p*<0.05). Small heterotrophic nanoflagellates were also included for comparison since they presented the greatest silver grain areas (Fig. 7C), yet they did not show significantly different areas among light treatments (*p*>0.05).

The uptake of ^3^H-leucine by *Thalassiosira* spp. at station AR4 was negatively affected by full sunlight (Fig. 7D, *p*<0.05), whereas no significant differences between the light treatments and the dark controls were observed for *Pseudonitzschia* spp. At station AN1, exposure to sunlight consistently inhibited their activity, but only *Thalassiosira* (sp. B) showed a clear negative effect of UVB compared to PAR+UVA exposure (Fig. 7E, *p*<0.05).

### Relative Contribution of Algal Groups and Bacteria to Substrate Uptake

**Figure 8:**
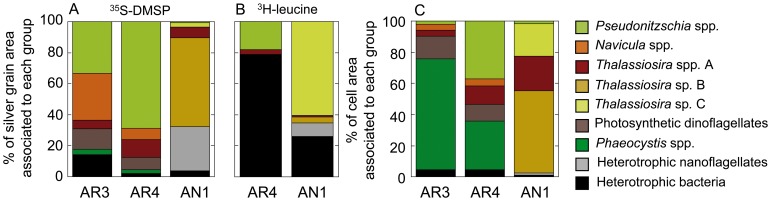
Estimated contribution of different organisms to total assimilation and biomass. Average relative contribution of each of the analyzed groups to the total silver grain area (as % of the sum of the silver grain area associated with all the considered groups) for ^35^S-DMSP (A) or ^3^H-leucine samples (B). (C) Relative contribution of each of the analyzed groups to total phytoplankton biomass (as % of the sum of cell areas of all the individuals of the considered groups). All estimates are averages of the three treatments.

The group-specific responses were calculated as the mean silver grain area around each group cells multiplied by the abundance of its active cells, and divided by the total sum of silver grain areas of the groups considered for image analysis. The patterns shown in Fig. 7 imply different relative contributions to total measured silver grain area among stations (Fig. 8A, B) and treatments (Table 3). This summed silver grain area can be considered as a proxy for the total algal uptake of ^35^S-DMSP or ^3^H-leucine provided that linearity between silver grain area and bulk incorporation is hold over time (see Discussion).

**Table 3:** Relative contribution of the analyzed groups to the total silver grain area after exposure to the different light treatments.

Relative contribution of each group to total analyzed silver grain area under different light treatments								
		Stn. AR3	Stn. AR4				Stn. AN1	
		DMSP	DMSP	Leucine			DMSP	Leucine
***Pseudonitzschia*** ** spp.**	DARK	16.9	48.4	72.0	***Pseudonitzschia*** ** spp.**	DARK	0.02	0.1
	PAR+UVA	59.0	80.7	89.5		PAR+UVA	1.2	0.0
	PAR+UVR	39.5	81.8	93.0		PAR+UVR	0.9	0.0
***Navicula*** ** spp.**	DARK	29.3	9.7	–	***Thalassiosira*** ** sp. A**	DARK	8.9	2.1
	PAR+UVA	33.0	6.8	–		PAR+UVA	7.2	0.7
	PAR+UVR	43.6	5.9	–		PAR+UVR	4.8	1.1
***Thalassiosira*** ** spp.**	DARK	8.9	15.2	28.0	***Thalassiosira*** ** sp. B**	DARK	47.6	8.2
	PAR+UVA	3.5	8.4	10.5		PAR+UVA	62.0	5.0
	PAR+UVR	7.9	11.1	7.0		PAR+UVR	68.3	3.1
**Photosynthetic dinoflagellates**	DARK	33.3	19.8	–	***Thalassiosira*** ** sp. C**	DARK	2.8	76.9
	PAR+UVA	4.6	3.9	–		PAR+UVA	3.5	83.0
	PAR+UVR	8.9	1.2	–		PAR+UVR	2.9	83.8
***Phaeocystis*** ** spp.**	DARK	11.7	7.0	–	**Heterotrophic nanoflagellates**	DARK	40.6	12.7
	PAR+UVA	0.0	0.2	–		PAR+UVA	26.1	11.3
	PAR+UVR	0.0	0.0	–		PAR+UVR	23.1	12.0

Percentages were calculated relative to the sum of the silver grain areas associated with all the groups considered for image analysis (excluding bacteria).

Additionally, an estimated contribution of each group to total biomass was calculated by multiplying the average cell area by the abundance of each group divided by the total sum of analyzed cell areas (Fig. 8C). Among these groups, Arctic *Pseudonitzschia* spp. accounted for a significant (stn. AR3) or even dominant (stn. AR4) contribution to total incorporation of ^35^S-DMSP or ^3^H-leucine, especially upon light exposure (Figs. 8A, B, Table 3), which was much higher than expected based on their relative abundances (Fig. 8C). Consequently, the contribution of the rest of the groups decreased under PAR+UVA or UVR and PAR +UVA conditions. The less active *Pseudonitzschia* at AN1 contributed only marginally to the uptake of either substrate (Fig. 8A, Table 3). At the Arctic stations, although the specific uptake of ^35^S-DMSP by *Phaeocystis* sp. was low (or totally absent under light incubations), its high abundance resulted in a substantial contribution (∼7–12%) to total uptake in the dark (Figs. 8A, C, Table 3). At station AN1, the low contribution of heterotrophic nanoflagellates to total cell biomass (<2%) contrasted with their high representation among the considered ^35^S-DMSP- or ^3^H-leucine-assimilating cells (up to 30% or 10%, respectively, Fig. 8A, B). The highly labeled, large *Thalassiosira* sp. C cells were responsible for most of the ^3^H-leucine silver grain area around algae (Fig. 8B), even more so under UVR exposure (Table 3). It must be noted, though, that the presence of heavily labeled large diatoms that could not be quantified for uptake, such as *Chaetoceros* spp. or *Eucampia* spp., suggests that the relative contributions of the counted organisms to total ^3^H-leucine uptake shown in Fig. 8B are most likely overestimates.

### Bacterial Contribution to Substrate Assimilation

In an attempt to estimate the contribution of heterotrophic bacteria to the uptake of the >5 µm fraction, we also quantified the silver grain area associated to the filter-retained prokaryotes. In general, their contribution to ^35^S-DMSP uptake was small (station AR3) to negligible (AR4 and AN1, Table 4, Fig. 8A) compared to that of the considered algal groups together. Conversely, their contribution to ^3^H-leucine uptake was as high as 79% and 26% at stations AR4 and AN1, respectively (Table 4), yet, as abovementioned, these latter values are overestimates because not all eukaryotic algae could be measured for their silver grain areas.

**Table 4:** Comparison between the relative contribution of prokaryotic and phytoplankton cells within 5 µm filters.

	Relative contribution to total silver grain area			
	(% of the sum of silver grain areas associated with all analyzed groups)			
	^35^S-DMSP		^3^H-Leucine	
	Prokaryotic cells	Phytoplankton cells	Prokaryotic cells	Phytoplankton cells
Stn. AR3	14.3	85.7	–	–
Stn. AR4	2.1	97.9	78.9	21.1
Stn. AN1	3.9	96.1	26.0	74.0

Percentages were calculated relative to the sum of silver grain areas associated to the analyzed algal groups plus heterotrophic bacteria, and values are means of the three treatments.

## Discussion

The osmotrophic uptake of organic compounds is believed to supplement autotrophic energy gain in algae, particularly in deep waters, systems with high allochthonous inputs or polar areas where algae need to survive during the long aphotic winter [1], [21], [22]. However, the spread and importance of DOM uptake by phytoplankton in natural marine communities and how it is influenced by natural radiation conditions still remain unclear. Our results show for the first time a direct effect of natural PAR and UVR levels on the uptake of ^3^H-leucine and ^35^S-DMSP by different phytoplankton taxa. Moreover, they provide the second evidence of ^35^S-DMSP uptake by natural marine eukaryotic phytoplankton assemblages, after Vila-Costa et al. [15].

### ‘Algal’ Versus ‘bacterial’ Assimilation of ^3^H-leucine and ^35^S-DMSP

Across the regions studied, the >5 µm fraction showed a frequent dominance of the assimilation of ^3^H-leucine and ^35^S-DMSP. This unexpected finding, which is opposite to the commonly found dominance of the smallest size fractions (mainly bacterioplankton) in the uptake of organic compounds [25], [35], points to a potentially important role of polar eukaryotic phytoplankton as low molecular weight (LMW)-DOM consumers.

The uptake of both leucine and DMSP was initially thought to be specific for heterotrophic bacteria [36], [37], yet several studies have demonstrated that different algal and cyanobacterial species can take up and assimilate either leucine [11], [22] or reduced sulfur from DMSP [15], [38], [39]. Algae are known to use different amino acids as carbon and nitrogen sources or to meet their cellular nitrogen demands, particularly when inorganic nitrogen is scarce [14], [40]. But very little is still known about the magnitude of algal DMSP assimilation in natural communities, its ecophysiological function, and how it influences the cycling of organic sulfur in the surface ocean and whether it can potentially regulate the emissions of volatile sulfur to the atmosphere.

### Sunlight Effects on the Bulk Assimilation by Organisms >5 µm

When samples amended with ^3^H-leucine were incubated under different light treatments, a general decrease in the incorporation by the >5 µm size fraction was observed towards full spectrum conditions. Interestingly, this inhibition was correlated with the UVR doses received by samples during incubations, suggesting that, similarly to what is observed for bacteria [41], the osmotrophic uptake of leucine by phytoplankton is negatively affected by solar radiation. Several experiments have also shown light-driven effects on the uptake of leucine by phytoplankton cells, yet depending on the species tested, either photostimulation or photoinhibition of uptake or consumption were reported [10], [21]. However, none of these experiments considered UVR exposure. On the other hand, the observed changes in ^35^S-DMSP assimilation are in accordance with the light-driven enhancement of this activity reported elsewhere for diatoms and picophototrophs [15], [38]. Unlike for leucine, the light-driven effects on DMSP assimilation were not related to irradiance levels, suggesting that DMSP-sulfur assimilation may be indirectly influenced by more complex, yet still poorly known, light-driven DMSP production, release and consumption dynamics (e.g. [42], [43]). In particular, the described antioxidant function of DMSP [44] might partially explain the observed light driven increases in its accumulation, yet it remains to be tested whether this function can be attained through the uptake of DMSP besides intracellular production [43].

### Widespread Algal Uptake of Organic Compounds by Taxonomic Groups

Whereas size fractionation may lead to inaccurate estimates of phytoplankton DOM-uptake since it does not completely separate algae from bacterial aggregates, attached bacteria, detritus or protozoa, autoradiographic surveys permit the rapid screening of algal populations taking up specific substrates (e.g. [40], [45]). Microautoradiography of 5 µm filters revealed an unexpected widespread capacity to take up both ^3^H-leucine and ^35^S-DMSP among a variety of algal phyla, mainly diatoms and photosynthetic dinoflagellates. While many Arctic phytoplankton groups were intensely labeled for ^35^S, the taxa present at the Antarctic station AN1 displayed lower numbers of active cells with weaker silver grain areas. This high uptake of ^35^S-DMSP in the Arctic might be related to the high DMSP supply rates released by the blooming *Phaeocystis* sp. [46] compared to the lower DMSP concentrations found in the sampled Antarctic waters (M. Galí et al., unpublished). Supporting this idea, Vila-Costa et al. [15] found higher numbers of Mediterranean ^35^S-assimilating diatoms in summer, when DMSP comprised a larger share of total sulfur and carbon fluxes. Accordingly, low- or non-DMSP producing diatoms (e.g. *Pseudonitzschia* spp.) would consume DMSP released by their high producing phytoplankton counterparts such as *Phaeocystis* sp. Should DMSP uptake supply energy, carbon or sulfur for growth, a DMSP-rich environment like summer Arctic waters might favor algal species capable of utilizing this substrate.

At the Antarctic station AN1, conversely, weak silver grain areas were usually observed for ^35^S-DMSP. Instead, great numbers of big diatoms (*Chaetoceros* spp., *Eucampia* spp., *Thalassiosira* spp.) appeared intensely labeled for ^3^H-leucine uptake. Finally, at station AN2, most of the signal was associated with the bacteria retained on the 5 µm filter and none of the blooming *Pseudonitzschia* appeared labeled for leucine.

Altogether, our findings highlight the helpfulness of single-cell resolution techniques to avoid misinterpretation of bulk assimilation data, and suggest that the osmotrophic uptake by phytoplankton should be considered when bulk bacterial activity measurements are conducted and interpreted. Since bacterial activity assays are generally performed in the dark, and darkness consistently led to the greatest uptake of ^3^H-leucine by phytoplankton, bacterial production may be overestimated depending on the abundance and activity of co-occurring phytoplankton.

It is not clear whether microautoradiography reflects actual assimilation (incorporation of the radiolabel into macromolecules) or just uptake. Organic molecules may enter a cell but fail to be metabolized at all, in which case they just accumulate in the cytoplasm [16], [47]. Samples were fixed with PFA after exposure to the radioisotopes, a process believed to cause cells to loose cytoplasm [48], so the fact that the autoradiographic signal remained after fixation points to substantial substrate assimilation. Moreover, the specific labeling patterns observed for some diatoms, which showed silver grains of ^3^H-leucine specifically associated with structures such as chloroplasts and nucleus, suggests incorporation of the amino acid into cellular macromolecules rather than simple uptake (see arrows in figs. 6B and D).

### Inter-group Variability in Algal Responses to Sunlight

In the microautoradiograms of algal samples subjected to image analysis, the differences in the silver grain area around cells were considered to reflect the effects of natural sunlight on the assimilation of these two organic compounds. The previously demonstrated linear relationship between silver grain areas and bulk incorporation of ^3^H-leucine allows comparison provided that incubation and exposure conditions are kept the same [33]. However, when organisms are very active, this linearity may be lost since after a while silver grain areas become denser but not larger, a fact that cannot be always detected by image analysis. In complex samples like ours, it is possible that some groups displayed higher uptake velocities than others and that this linearity was not maintained over time for all of them. Therefore, our results must be regarded as trends and general patterns, but not as absolute values or incorporation rates. In any case, the lack of linearity would lead to reduced differences among groups or treatments, or a misdetection of the whole range of variation, but not to an artifactual creation of the observed differences.

Quantification of the silver grain areas around cells revealed a variety of group-specific responses to sunlight which further varied depending either on the substrate or the station considered. Taken together, it appeared that natural irradiance levels were significantly influencing the osmoheterotrophic activity of the studied phytoplankton assemblages. However, since a given group responded differently depending on the substrate analyzed (i.e., photostimulation for ^35^S-DMSP and photoinhibition for ^3^H-leucine) these results cannot be solely considered as an indication of an inhibitory effect of UVR on the activity of the organism. Besides the potentially differential damage of UVR onto uptake systems [49], several other mechanisms have been proposed for the observed light-driven effects on DOM uptake by phytoplankton. Algal utilization of amino acids has been shown to be most significant in the absence of photosynthesis, e.g., in turbid waters, in dark incubations or at night [10], [50], [51], [52]. It has been suggested that, during the day, the products of photosynthesis and the uptake of nitrate, ammonium or urea restrict the uptake of amino acids by increasing the intracellular amino acid pool [51]. A lower uptake in the light could also be due to dilution of the labeled substrate with newly photosynthesized substrate, or to transport systems under repression by photosynthesis catabolites [6]. Thus, factors other than the light conditions, such as the natural substrate concentration and maybe the past environmental history of the algae affect each species’ ability to assimilate organic substrates.

Our observation that ^35^S-DMSP uptake by various algal groups is enhanced by light could suggest that the photosynthetic apparatus harvests light and transfers this energy into ATP that is used for supplementary powering the active uptake of DMSP. Alternatively, an increased DMSP release by UVR-stressed algae [43], [44] might activate the uptake systems of their low DMSP-producing counterparts. Finally, the potential use of DMSP as an antioxidant [43] might explain its accumulation under light conditions. In any case, this light-driven algal DMSP uptake (also observed in the size fractionated assimilation) would lead to higher shares in the hitherto overlooked contribution of eukaryotic phytoplankton as a DMSP sink, particularly in the long daylight of the polar summer.

### Relative Contribution of Algal Groups and Bacteria to Substrate Uptake

In spite of the aforementioned limitations of the method, image analysis of silver grain and cellular areas allowed a rough estimation of each group’s contribution to the measured substrate uptake. As seen by the different patterns shown by figures 8A, B and C, this contribution was hardly ever related to the group’s contribution to biomass, highlighting a diversity of roles in phytoplankton osmoheterotrophic activity that cannot be predicted from their abundances alone. This, joined to each group’s differential responses to sunlight, suggests that solar radiation may modulate not only the quantity but also the direction of the fluxes of some organic compounds through the different compartments of the microbial food webs.

The silver grain areas associated with 5 µm-retained bacteria were also quantified in order to estimate their contribution to the substrate assimilation regarded as ‘algal’. On average, retained bacteria comprised <4% of the total bacterial abundance in the Arctic waters and 9–16% in the Antarctic stations [32]. Altogether, our results suggest that phytoplankton were responsible for the vast majority of the ^35^S-DMSP assimilation in the 5 µm fraction, whereas these aggregated or attached bacteria contributed a major proportion of ^3^H-leucine assimilation (AR3, AR4 and AN2 samples), except at station AN1 where big diatoms labeled for ^3^H-leucine were abundant. In any case, since not all phytoplankton groups were analyzed, these observations are most likely overestimates of the interfering role of associated bacteria in leucine assimilation by the algal fraction.

It has been proposed that sunlight has the potential to favor picophytoplankton in their competition for DMSP uptake against heterotrophic bacteria, as observed for the Mediterranean cyanobacterium *Synechococcus* [39]. Since free-living heterotrophic bacteria from the same polar stations mostly showed non-significant or negative light-driven effects in their number of ^35^S-labelled cells (see Table 4 in [32]), it is likely that the observed photostimulation of certain algal groups would also lead to an increased competition of eukaryotic phytoplankton for total DMSP uptake relative to that of bacteria. In view of the differential responses and substrates affinities, though, this process will be strongly dependent on the identity of the organisms involved.

Our findings confirm a major role of solar radiation on DMSP dynamics in the ocean [53], [54], but they unveil a new mechanistic twist: depending on the microbial consortia, light levels may favor DMSP-sulfur uptake by a fraction of the eukaryotic phytoplankton assemblage, thus diverting DMSP from being further catabolized into volatile DMS. Thus, sunlight simultaneously favors [55] and hampers [this work] DMS production, inhibits microbial DMS consumption and destroys DMS through photolysis [56]. This DMS cycle is a paradigmatic example of how sunlight and biogeochemical processes in the pelagic ocean are so intimately entangled in processes and counter-processes that result in largely buffered dynamics [57].

Part of the radioisotope incorporation by algae could have occurred through bacterivory or phagotrophy. Members of the dinophytes, cryptophytes and haptophytes have been shown to feed on bacteria or other algae [17], [18], [58] whereas diatoms do not. Diatoms, particularly polar species that have to survive long winter darkness, are known for their dark survival potentials [59], [60], [61] with facultative heterotrophy as one of their strategies. Therefore, since diatoms were major contributors to ^35^S-DMSP uptake in the studied stations (Fig. 8A), an important fraction of the substrate uptake must have occurred by osmoheterotrophy rather than phagotrophy. Yet, uptake by non-diatom ingestion of ^35^S- or ^3^H-labeled bacteria or small algae cannot be totally discarded, and bacterivory can also be regulated by light [62], [63]. Small heterototrophic nanoflagellates at station AN1 could have been grazing on bacteria and, interestingly, their responses to light were similar to those of particular bacterial groups from this station labeled for the same substrates (see Figs. 3 and 4 in [32]). Conversely, large hetero- or phototrophic Arctic dinoflagellates such as *Protoperidinium* spp. (Fig. 5B), *Prorocentrum* spp. and *Leucocryptos marina* appeared intensely labeled for ^35^S-DMSP but not for ^3^H-leucine. This suggests that either osmotrophic uptake [64] or grazing on other algae, but not bacterivory, was a major source of the ^35^S label found in these other groups.

Overall, our findings support the notion of a major and widespread heterotrophic activity within phytoplankton assemblages in summer Arctic and Antarctic waters. The use of autoradiography combined with size-fractionated assimilation offers a way to screen mixed phytoplankton populations for heterotrophic potential, revealing distinct affinities and behavioral trends in polar algae with regard to ^3^H-leucine and ^35^S-DMSP uptake and solar radiation. Future experiments combining different incubation times with measurements of assimilation and silver grain areas should allow a more quantitative assessment of this phenomenon, a necessary step to introduce algal osmoheterotrophy into elemental budget and numerical simulations of the planktonic food web.
